# Investigating the associations between lumbar paraspinal muscle health and age, BMI, sex, physical activity, and back pain using an automated computer-vision model: a UK Biobank study

**DOI:** 10.1016/j.spinee.2024.02.013

**Published:** 2024-07

**Authors:** Evert Onno Wesselink, Annelies Pool-Goudzwaard, Benjamin De Leener, Christine Sze Wan Law, Meredith Blair Fenyo, Gabriella Marie Ello, Michel Willem Coppieters, James Matthew Elliott, Sean Mackey, Kenneth Arnold Weber

**Affiliations:** a Faculty of Behavioural and Movement Sciences, Amsterdam Movement Sciences, Vrije Universiteit Amsterdam, Van der Boechorststraat 9, 1081 BT Amsterdam, The Netherlands; b Division of Pain Medicine, Department of Anaesthesiology, Perioperative and Pain Medicine, Stanford University, 1070 Arastradero Road, Palo Alto, CA 94304, USA; c SOMT University of Physiotherapy, Softwareweg 5, 3821 BN Amersfoort, the Netherlands; d Department of Computer Engineering and Software Engineering, Polytechnique Montreal, 2900 Edouard Montpetit Blvd, Quebec H3T 1J4, Canada; e School of Health Sciences and Social Work, Menzies Health Institute Queensland, Griffith University, Brisbane and Gold Coast, 170 Kessels Road, 4111 Brisbane, Australia; f The University of Sydney, Faculty of Medicine and Health and the Northern Sydney Local Health District, The Kolling Institute, Reserve Road, St Leonards NSW Sydney 2065, Australia

**Keywords:** Adipose tissue, Artificial intelligence, Back muscles, Fatty infiltration, Low back pain, Magnetic resonance imaging

## Abstract

**Background context:**

The role of lumbar paraspinal muscle health in back pain (BP) is not straightforward. Challenges in this field have included the lack of tools and large, heterogenous datasets to interrogate the association between muscle health and BP. Computer-vision models have been transformative in this space, enabling the automated quantification of muscle health and the processing of large datasets.

**Purpose:**

To investigate the associations between lumbar paraspinal muscle health and age, sex, BMI, physical activity, and BP in a large, heterogenous dataset using an automated computer-vision model.

**Design:**

Cross-sectional study.

**Patient sample:**

Participants from the UK Biobank with abdominal Dixon fat-water MRI (N=9,564) were included (41.8% women, mean [SD] age: 63.5 [7.6] years, BMI: 26.4 [4.1] kg/m^2^) of whom 6,953 reported no pain, 930 acute BP, and 1,681 chronic BP.

**Outcome measures:**

Intramuscular fat (IMF) and average cross-sectional area (aCSA) were automatically derived using a computer-vision model for the left and right lumbar multifidus (LM), erector spinae (ES), and psoas major (PM) from the L1 to L5 vertebral levels.

**Methods:**

Two-tailed partial Pearson correlations were generated for each muscle to assess the relationships between the muscle measures (IMF and aCSA) and age (controlling for BMI, sex, and physical activity), BMI (controlling for age, sex, and physical activity), and physical activity (controlling for age, sex, and BMI). One-way ANCOVA was used to identify sex differences in IMF and aCSA for each muscle while controlling for age, BMI, and physical activity. Similarly, one-way ANCOVA was used to identify between-group differences (no pain, acute BP, and chronic BP) for each muscle and along the superior-inferior expanse of the lumbar spine while controlling for age, BMI, sex, and physical activity (α=0.05).

**Results:**

Females had higher IMF (LM mean difference [MD]=11.1%, ES MD=10.2%, PM MD=0.3%, p<.001) and lower aCSA (LM MD=47.6 mm^2^, ES MD=350.0 mm^2^, PM MD=321.5 mm^2^, p<.001) for all muscles**.** Higher age was associated with higher IMF and lower aCSA for all muscles (r≥0.232, p<.001) except for LM and aCSA (r≤0.013, p≥.267). Higher BMI was associated with higher IMF and aCSA for all muscles (r≥0.174, p<.001). Higher physical activity was associated with lower IMF and higher aCSA for all muscles (r≥0.036, p≤.002) except for LM and aCSA (r≤0.010, p≥.405). People with chronic BP had higher IMF and lower aCSA than people with no pain (IMF MD≤1.6%, aCSA MD≤27.4 mm^2^, p<.001) and higher IMF compared to acute BP (IMF MD≤1.1%, p≤.044). The differences between people with BP and people with no pain were not spatially localized to the inferior lumbar levels but broadly distributed across the lumbar spine.

**Conclusions:**

Paraspinal muscle health is associated with age, BMI, sex, and physical activity with the exception of the association between LM aCSA and age and physical activity. People with BP (chronic>acute) have higher IMF and lower aCSA than people reporting no pain. The differences were not localized but broadly distributed across the lumbar spine. When interpreting measures of paraspinal muscle health in the research or clinical setting, the associations with age, BMI, sex, and physical activity should be considered.

## Introduction

Back pain (BP) is the leading cause of disability worldwide with a lifetime prevalence up to 84% and an annual prevalence between 22% and 65% [1]. Despite persistent efforts to improve the management of BP, including sophisticated diagnostic techniques and novel treatments, the global burden of BP continues to grow [1]. The development of BP is multifactorial, driven by the interaction between many biological, psychological, and social factors [2].

In this study, we seek to advance our understanding of one of these biological factors contributing to BP, namely the role of lumbar paraspinal muscle health. Decline in paraspinal muscle health in BP has been characterized by increased intramuscular fat (IMF) and decreased size (ie, muscle atrophy) [3]. While researchers have hypothesized that paraspinal muscle health decline plays an important role in spinal function [3], the literature is inconclusive about the magnitude and meaning of these changes in people with BP. In some studies, increases in IMF and decreases in the size of the lumbar paraspinal muscles were highly associated with the presence and severity of BP [4], although others disagree [5]. The inconsistency in the literature may be related to the accuracy and reliability of the methods used to assess muscle health, not adequately accounting for confounding factors, and the use of small samples sizes providing low statistical power [6].

Paraspinal muscle health decline in BP is complex. Ongoing effects of pain, inactivity, and inflammation [7] as well as different time-dependent structural remodeling mechanisms may influence this decline [7]. However, paraspinal muscle health decline is not specific to people with BP and even seen in asymptomatic individuals [8]. Paraspinal muscle health decline may vary locally by segmental level [9] and may be masked by other factors (eg, age, body mass index (BMI), sex, and physical activity) [8]. As most studies have investigated small cohorts of BP participants, the studies have lacked variability in sample characteristics indicating the need for a broad assessment of lumbar paraspinal muscle health in a large, heterogenous dataset.

Muscle composition and size can be quantified noninvasively using conventional (T_1_ and T_2_-weighted) and advanced (Dixon and proton density fat fraction) magnetic resonance imaging (MRI). The assessment of muscle health with MRI has traditionally required manual segmentation of the muscle boundaries. Manual segmentation inefficiencies have impeded using these methods in large-scale studies and clinical practice [10]. Applying advanced computer-vision approaches to MRI, such as convolutional neural networks (CNN), has been transformative, enabling automated quantification of muscle health with high efficiency and human-level performance [10,11]. This combined with the recent availability of large, heterogenous whole-body imaging datasets, such as the UK Biobank, provides both the tools and data to transform our understanding of the relationship between muscle health and BP.

To improve our understanding of lumbar paraspinal muscle health, we investigated the associations between lumbar paraspinal muscle health and age, BMI, sex, physical activity, and BP using an automated computer-vision model and Dixon fat-water MRI from 9,564 participants with and without BP from the UK Biobank. We assessed the differences between people with BP and no pain both globally and locally along the superior-inferior expanse of the lumbar spine. We hypothesized that paraspinal IMF and muscle size would be associated with age, BMI, sex, physical activity, and BP. Furthermore, we hypothesized that the differences between people with BP and no pain would be most profound at the lower lumbar spine, where spinal pathology is most commonly reported.

## Methods

### UK Biobank

The UK Biobank is a large, on-going prospective cohort study, established primarily to investigate the genetic and lifestyle determinants of various diseases of middle and later life [12]. The UK Biobank received approval from the National Information Governance Board for Health and Social Care and the National Health Service Northwest Multicentre Research Ethics Committee. The Strengthening the Reporting of Observational Studies in Epidemiology reporting guideline for cross-sectional studies were followed [13].

In this cross-sectional study, we used the initial imaging assessment responses (application number 67450) to identify cohorts of participants with no pain, acute BP (ie, less than 3 months), and chronic BP (ie, more than 3 months). Participants were asked: “In the last month, have you experienced any of the following that interfered with your usual activities?” (Data-Field 6159). Participants experiencing BP were then asked about the duration of the BP: “Have you had back pains for more than 3 months?” (Data-Field 3571). The no pain cohort was identified by a response of *none of the above* to pain experienced over the month prior, the acute BP cohort was identified by a response of *yes* to BP in the last month and *no* to BP lasting longer than 3 months, and the chronic BP cohort was identified by a response of *yes* to BP in the last month and *yes* to BP lasting longer than 3 months. No additional clinical pain measures regarding BP were accessible with the initial imaging assessment. Self-reported physical activity was assessed using the Short International Physical Activity Questionnaire (IPAQ), which encompasses the frequency, intensity, and duration of walking, moderate, and vigorous activities [14]. The time devoted to walking, moderate, and vigorous activities was adjusted based on the estimated energy expended in each activity category. This adjustment was made to calculate the Metabolic Equivalent of Task (MET) minutes per week, representing the total physical activity [15].

### Dixon fat-water MRI

Dixon fat-water abdominal MRI was performed supine using a 1.5T Siemens Aera MR scanner (Syngo MR D13, Siemens, Erlangen Germany) (VIBE, TE_1_=2.39 ms, TE_2_=4.77 ms, TR=6.69 ms, flip angle=10°, matrix size=224×174, in-plane resolution=2.232 mm×2.232 mm, slice thickness=4.500 mm). Fat and water magnitude images were reconstructed from the in-phase and out-of-phase acquisitions [16]. To assess muscle composition, we calculated IMF as the percent of the total signal (water+fat) attributed to fat. As height varies between sexes [17] and across the lifespan [17], we calculated each muscle's cross-sectional area and normalized the total CSA by the number of slices segmented to calculate the average cross-sectional area (aCSA).

### CNN training and testing

A blinded rater (EW) with extensive training in lumbar spine anatomy and imaging manually segmented the muscles of interest (ie, left and right lumbar multifidus, erector spinae, and psoas major) across the L1–L5 vertebral levels using anatomical cross-references as previously described [18]. This manual segmentation process was previously tested for its reliability and accuracy, demonstrating a high level of segmentation accuracy and excellent inter-rater reliability [10]. We determined that a total of 130 participants (65 participants with no pain and 65 participants with chronic BP) to be an optimal cut-off between the time-consuming burden of manual segmentation while providing sufficient clinical variability (ie, age, sex, BMI, physical activity, and BP status) in the training and testing datasets. The images were split into training (n=100, 47.0% chronic BP, 50.0% female, mean (SD) age: 63.1 (7.7) years, BMI: 26.0 (3.9) kg/m^2^) and testing datasets (n=30, 46.7% chronic BP, 53.3% female, age: 62.2 (7.9) years, BMI: 26.5 (3.5) kg/m^2^) and were matched for sex (p=.261), age (p=.593), BMI (p=.430), and BP status (p=.732). We trained a modified 2D U-Net CNN for image segmentation using the MONAI framework for deep learning in healthcare imaging (See Supplementary Methods for CNN training specifications) [19]. The CNN used in this study will be made openly available at https://github.com/MuscleMap/MuscleMap.

### Inclusion and exclusion criteria

Participants were excluded for withdrawal from the UK Biobank, not attending the initial imaging assessment, missing BMI, missing self-reported physical activity, self-reported history of cancer (Data-Field 20001), substance use disorder, other major medical conditions (Data-Fields 20001 and 20002), and no Dixon fat-water imaging. Next, a blinded rater (EW) performed a visual inspection of the images and excluded participants for inadequate field-of-view (ie, not capturing the L1 and L5 vertebral levels), presence of fat-water swapping artifacts, and poor image quality. The same blinded rater also visually inspected the CNN muscle segmentations and excluded participants based on poor segmentation quality.

### Statistical analyses

Two-tailed partial Pearson correlations were generated for each muscle to identify the direction and magnitude of linear relationships between the muscle measures (IMF and aCSA) and age (controlling for sex, BMI, and physical activity), BMI (controlling for age, sex, and physical activity), and physical activity (controlling for age, BMI, and sex) in people with no pain. Additionally, we used a one-way ANCOVA to identify sex differences in IMF and aCSA for each muscle while controlling for age, BMI, and physical activity. Similarly, a one-way ANCOVA was used to identify between-group differences (no pain, acute BP, and chronic BP) for each muscle while controlling for age, BMI, sex, and physical activity. To assess the spatial distribution of IMF and CSA and between-group differences in IMF and CSA along the superior-inferior expanse of the lumbar spine, we calculated IMF and CSA at each axial slice. Using spline interpolation (interp1d, SciPy Version 1.9.1), we normalized the IMF and CSA measures along the superior-inferior axis such that the most inferior and superior slices corresponded to 0% and 100% of the muscle length, respectively [20]. We then assessed IMF and CSA using a one-way ANCOVA at each axial slice (controlling for age, BMI, sex, and physical activity) to identify where IMF and CSA differed between the no pain, acute BP, and chronic BP groups for each muscle. Model residuals were tested for normality and sphericity using Skewness, Kurtosis, Shapiro–Wilk, Q–Q plots, and Mauchly's test of Sphericity. We used an α of 0.05 as the threshold for statistical significance, and Bonferroni correction to adjust the family-wise error rate for multiple comparisons. We performed statistical analyses in R-studio (Version 4.2.2).

## Results

From the 502,485 participants in the UK Biobank, 29,656 participants with imaging were included after initial screening, of which 15,597 were excluded based on inadequate field-of-view, 54 due to fat-water swapping artifacts, 43 due to poor image quality, and 71 due to poor segmentation quality (31% female, mean [SD] age: 62.3 [7.5] years, BMI: 35.2 [9.8] kg/m^2^, no pain: 20 [28.2%], acute BP 7 [9.9%], chronic BP 18 [25.4%], and no BP but other pain conditions 26 [36.6%]). From the remaining 13,891 participants, 6,953 participants with no pain (41.7% female, mean [SD] age: 63.7 [7.5] years, BMI: 26.1 [3.9] kg/m^2^, MET: 2,794.6 [2,359.7]), 930 with acute BP (37.2% female, age: 62.5 [7.6] years, BMI: 26.6 [4.1] kg/m^2^, MET: 2,852.3 [2,543.2]), and 1,681 with chronic BP (44.7% female, age: 63.2 [7.9] years, BMI: 27.4 [4.7] kg/m^2^, MET: 2,859.1 [2,711.1]) were included. The remaining 4,327 participants were excluded due to no reported BP but endorsing other pain conditions (Supplementary Fig. 1).

### Convolutional neural network

**Fig. 1:**
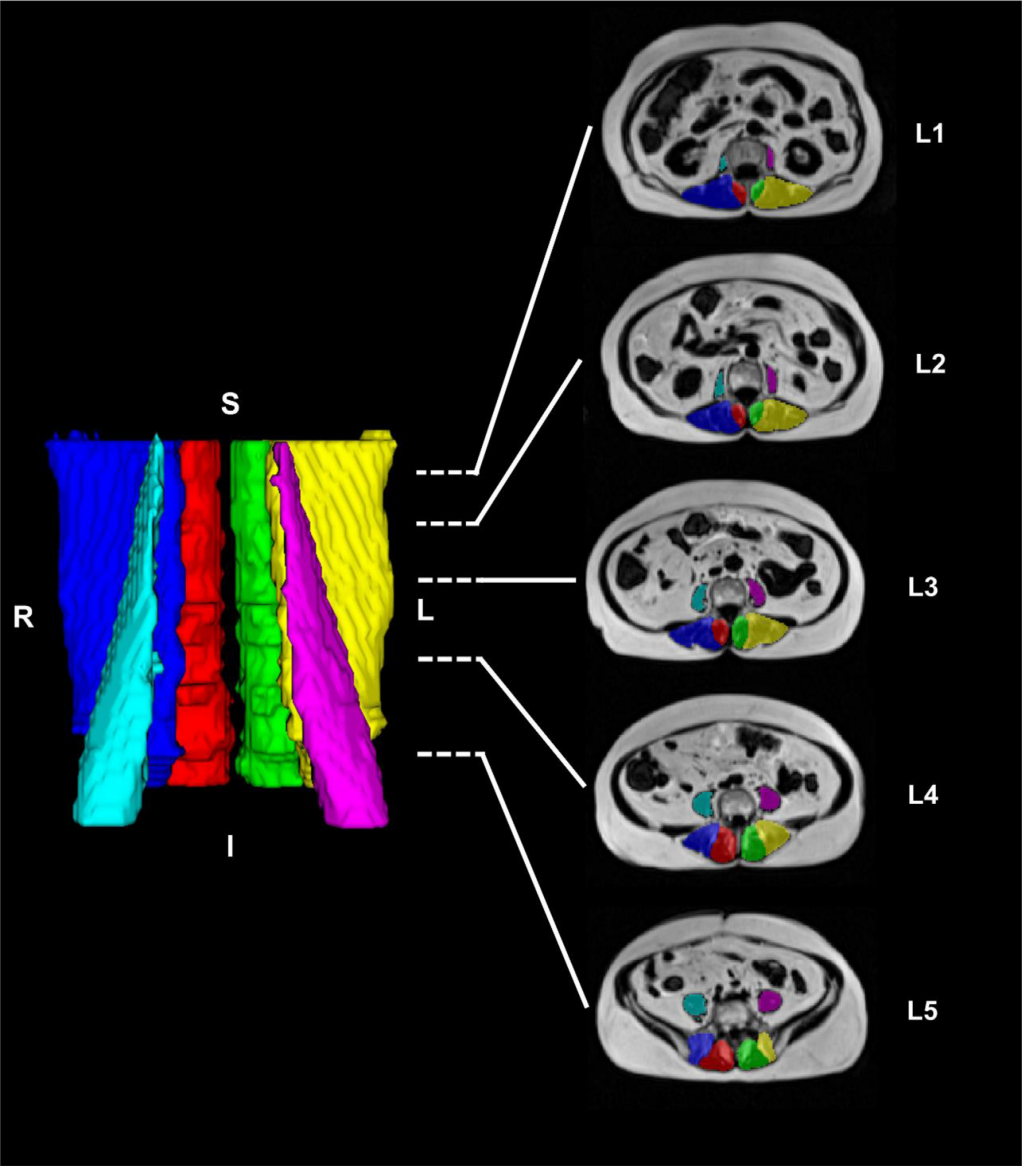
3D rendering (left) and 2D renderings at the L1-L5 vertebral levels (right) of the paraspinal muscle segmentations from the automated computer-vision model. Muscle segmentation masks of the right lumbar multifidus (red), left lumbar multifidus (green), right erector spinae (dark blue), left erector spinae (yellow), right psoas major (turquoise), and left psoas major (purple) are shown. *R* Right, *L* Left, *S* Superior, *I* Inferior.

Training of CNN was completed in 150,000 iterations. The trained CNN models segmented all axial slices in an image in 1.96 (0.03) seconds (Fig. 1), with high segmentation accuracy (Sørensen-Dice Index≥0.892) for the CNN for all muscles (Supplementary Table 1). Furthermore, we report high CNN accuracy and reliability for IMF (MAE≤0.919%, ICC_2,1_≥0.869) and aCSA (MAE≤11.39 mm^2^, ICC_2,1_≥0.949) (Supplementary Table 2). Bland-Altman and correlations plots are shown in Supplementary Figs. 2 and 3.

### Associations with age, BMI, sex, and physical activity in people with no pain

**Fig. 2:**
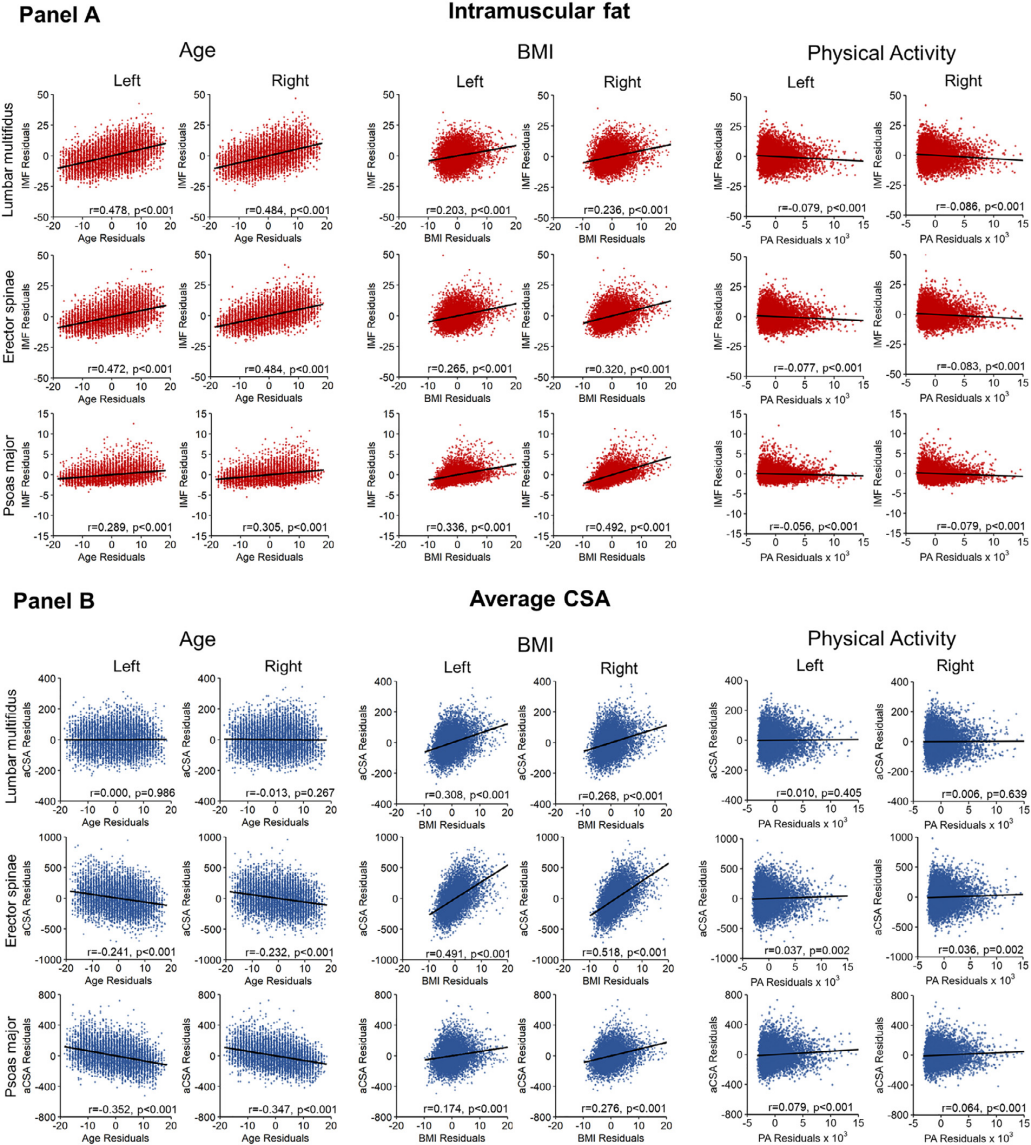
Associations between intramuscular fat (IMF) (Panel A) and average cross-sectional area (aCSA) (Panel B) and age, body mass index (BMI), and physical activity for the left and right lumbar multifidus, erector spinae, and psoas major in people with no pain. Partial correlations (Pearson's r) were performed to identify linear relationships between IMF or aCSA and age, BMI, and physical activity when controlling for age, sex, BMI, and/or physical activity, respectively (residuals plotted). Note: The y-axis is scaled differently for each muscle to better visualize the correlations.

**Fig. 3:**
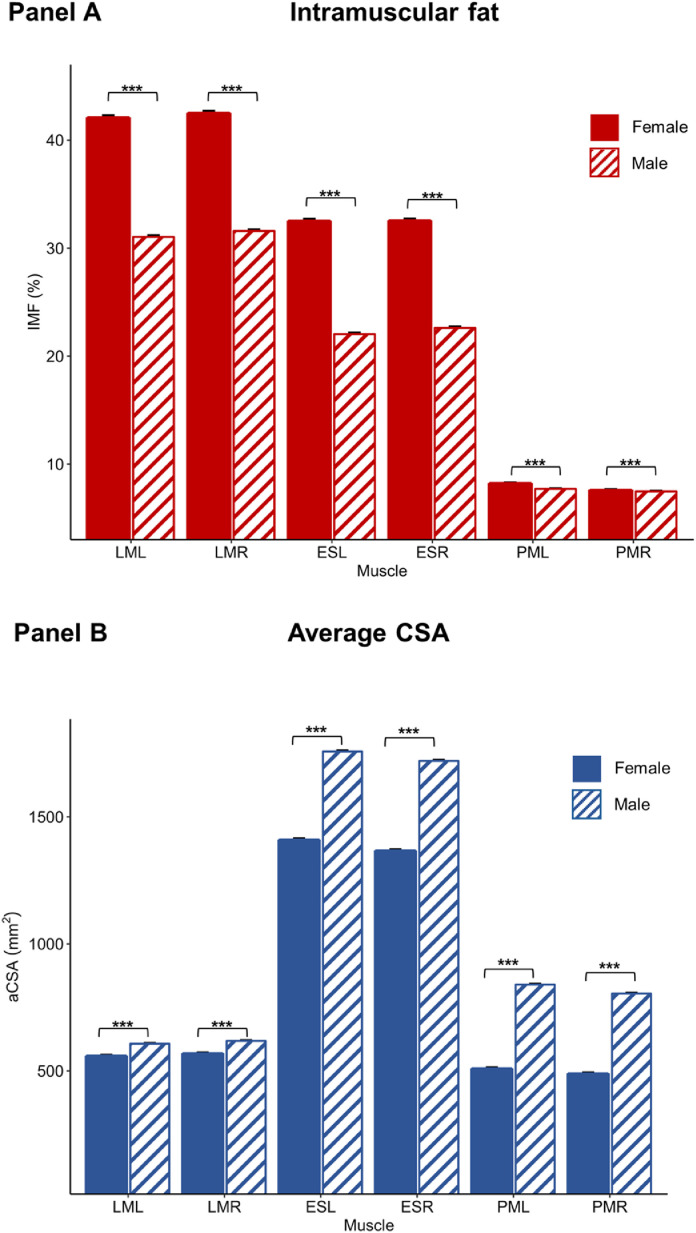
Estimated marginal means for IMF (Panel A) and aCSA (Panel B) by sex (controlling for age, BMI, and physical activity) for the left and right lumbar multifidus (LM), erector spinae (ES) and psoas major (PM). Females have higher IMF and lower aCSA for all muscles. Error bars=1 SE. *p<.05, **p<.01, ***p<.001.

Mean (SD) IMF was 36.2% (10.1) for the lumbar multifidus, 27.0% (9.3) for erector spinae, and 7.8% (1.7) for psoas major. Age and IMF (controlling for BMI, sex, and physical activity) were positively correlated for the lumbar multifidus (r≤0.484, p<.001), erector spinae (r≤0.484, p<.001), and psoas major (r≤0.305, p<.001). BMI and IMF (controlling for age, sex, and physical activity) were positively correlated for the lumbar multifidus (r≤0.236, p<.001), erector spinae (r≤0.320, p<.001), and psoas major (r≤0.492, p<.001). Physical activity and IMF (controlling for age, sex, and BMI) were negatively correlated for the lumbar multifidus (r≤0.086, p<.001), erector spinae (r≤0.083, p<.001), and psoas major (r≤0.079, p<.001) (Fig. 2). Females had higher IMF (controlling for age, BMI, and physical activity) for the lumbar multifidus (mean difference (MD)=11.1%), erector spinae (MD=10.2%), and psoas major (MD=0.3%) (p<.001) (Fig. 3).

Mean (SD) aCSA was 592.3 mm^2^ (84.2) for the lumbar multifidus, 1596.0 mm^2^ (288.3) for ES, and 686.5 mm^2^ (208.4) for psoas major. Age and aCSA (controlling for BMI, sex, and physical activity) were negatively correlated for the erector spinae (r≤0.241, p<.001) and psoas major (r≤0.352, p<.001) but not the lumbar multifidus (r≤0.013, p≥.267). BMI and aCSA (controlling for age, sex, and physical activity) were positively correlated for the lumbar multifidus (r≤0.308, p<.001), erector spinae (r≤0.518, p<0.001), and psoas major (r≤0.276, p<.001). Physical activity and aCSA (controlling for age, sex, and BMI) were positively correlated for the erector spinae (r≤0.037, p=.002) and psoas major (r≤0.079, p<.001) but not the lumbar multifidus (r≤0.010, p≥.405) (Fig. 2). Females had lower aCSA (controlling for age, BMI, and physical activity) for the lumbar multifidus (MD=47.6 mm^2^), erector spinae (MD=350.0 mm^2^), and psoas major (MD=321.5 mm^2^) (p<.001) (Fig. 3).

### Lumbar paraspinal muscle health between people with and without BP

**Fig. 4:**
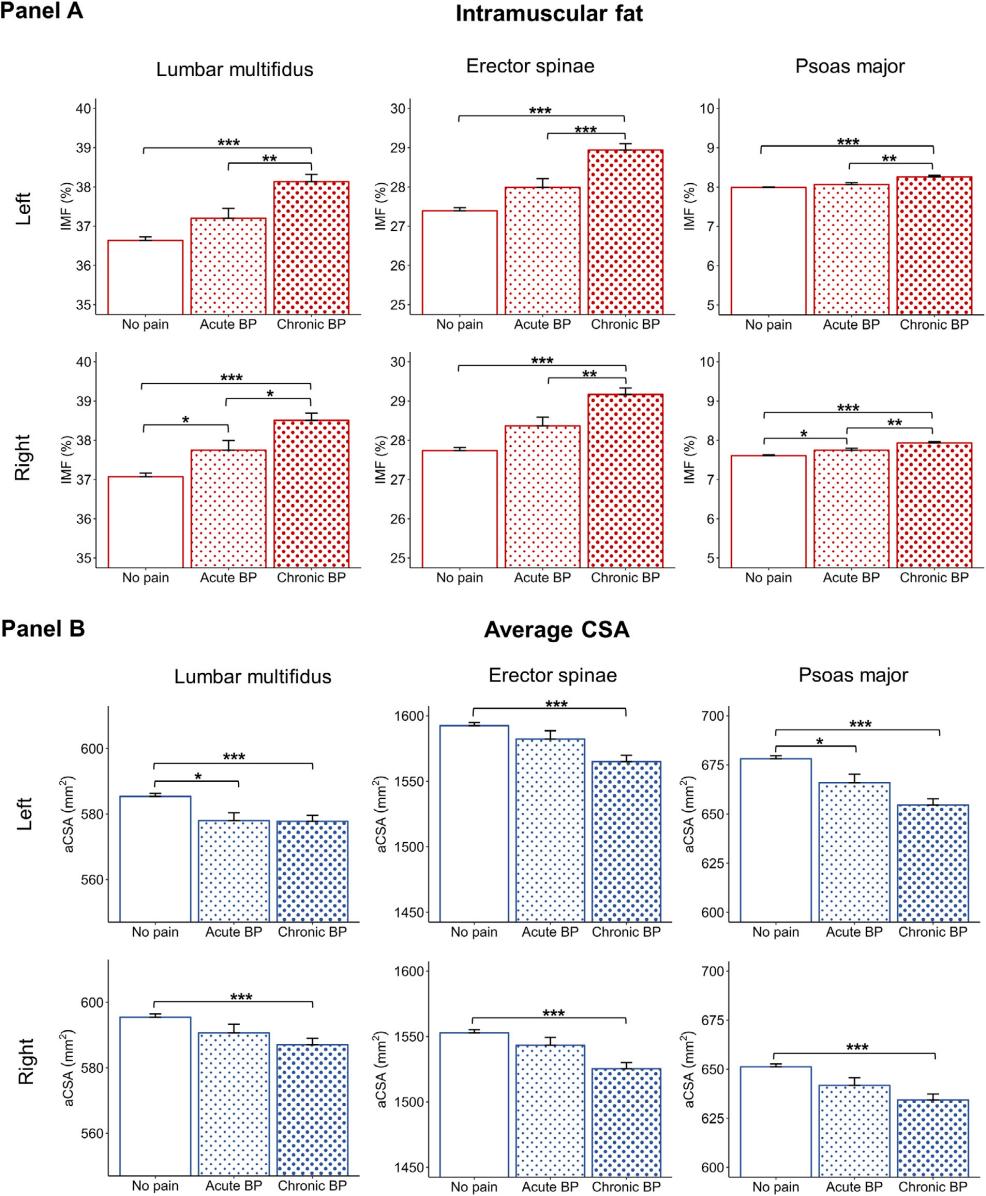
Between-group differences (mean±SE) in intramuscular fat (IMF) (Panel A) and average cross-sectional area (aCSA) (Panel B) for the left and right lumbar multifidus, erector spinae, and psoas major in participants with no pain, acute back pain (BP), and chronic BP. Estimated marginal means are shown for each group after controlling for age, BMI, sex, and physical activity. People with chronic BP had significantly higher IMF and lower aCSA for all muscles compared to people with no pain. Error bars=1 SE. Note: The y-axis is scaled differently for each muscle to better visualize the group differences. *p<.05, **p<.01, ***p<.001.

We found significant between-group differences (controlling for age, BMI, sex, and physical activity) for IMF for all muscles (p<.001). IMF was higher in people with chronic BP than in people with no pain (MD≤1.6%, p<.001) and acute BP (MD≤1.1%, p≤.044). IMF was higher in people with acute BP than in people with no pain for the right lumbar multifidus (MD=0.7%, p=.034) and the right psoas major (MD=0.1%, p=.038) (Fig. 4).

For aCSA, we found significant between-group differences for the erector spinae and psoas major (p<.001) but not for the lumbar multifidus (p≥.385) (while controlling for age, BMI, sex, and physical activity). aCSA was lower in people with chronic BP than in people with no pain for all muscles (MD≤27.4 mm^2^, p<.001). Between people with chronic BP and acute BP, no significant differences in aCSA were found for all muscles (MD≤17.8 mm^2^, p≥.058). aCSA was lower in people with acute BP than those without pain for the left lumbar multifidus (MD=7.4 mm^2^, p=.012) and left psoas major (MD=12.1 mm^2^, p=.022) (Fig. 4).

### Spatial distribution of lumbar paraspinal muscle health in people with and without BP

**Fig. 5:**
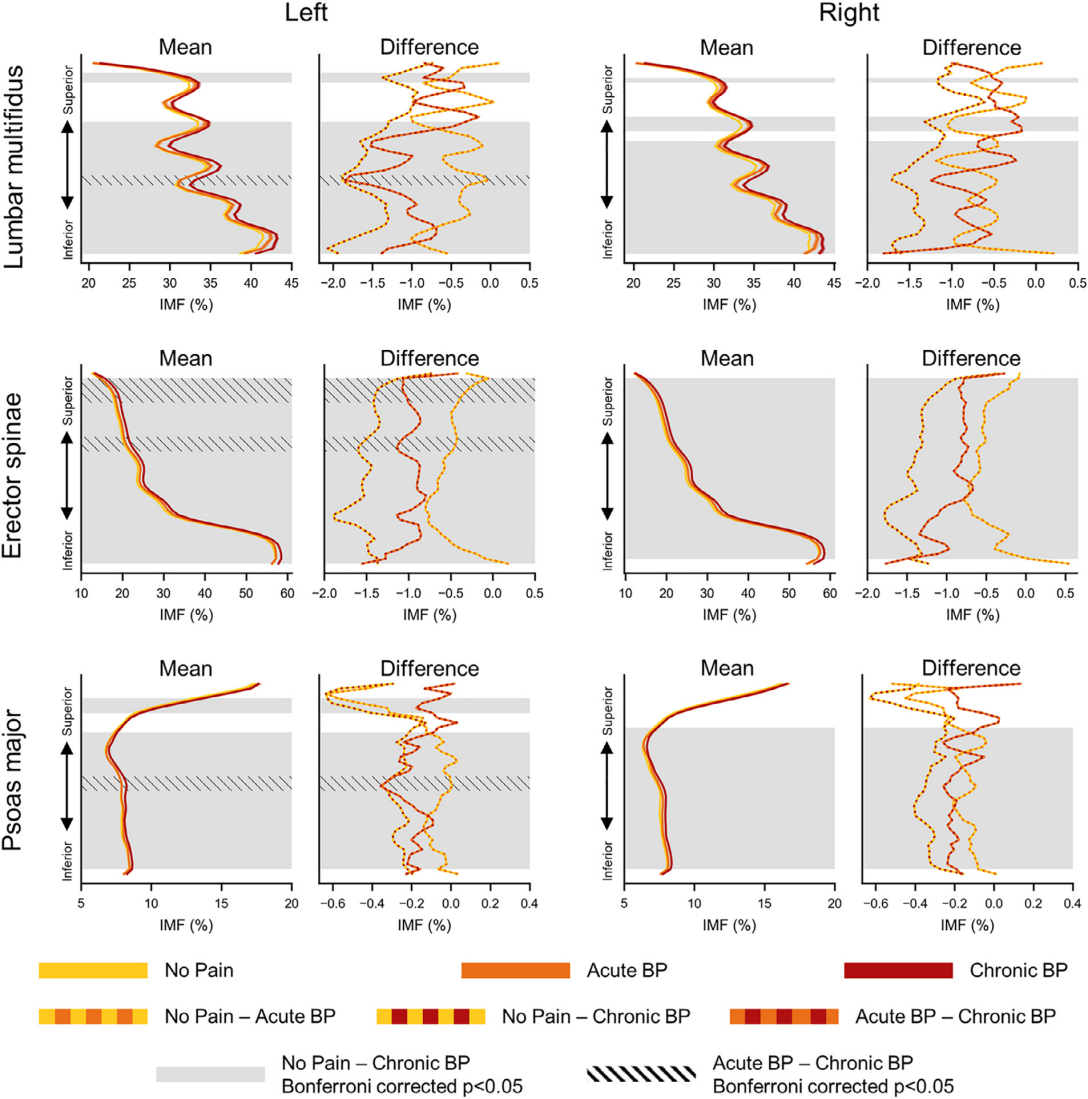
Spatial distribution of intramuscular fat (IMF) along the superior-inferior expanse of the lumbar spine for the left and right lumbar multifidus, erector spinae, and psoas major. Mean IMF (±1 SE error bands) for people with no pain, acute back pain (BP), and chronic BP (Mean) as well as between-group differences (Difference) in IMF are shown. We normalized the IMF measures along the superior-inferior axis such that the inferior most and superior most slices corresponded to 0% and 100% of the lumbar muscle length, respectively. Gray and hatched shading = Bonferroni corrected p-value <.05, showing significant between-group differences after family-wise error correction.

**Fig. 6:**
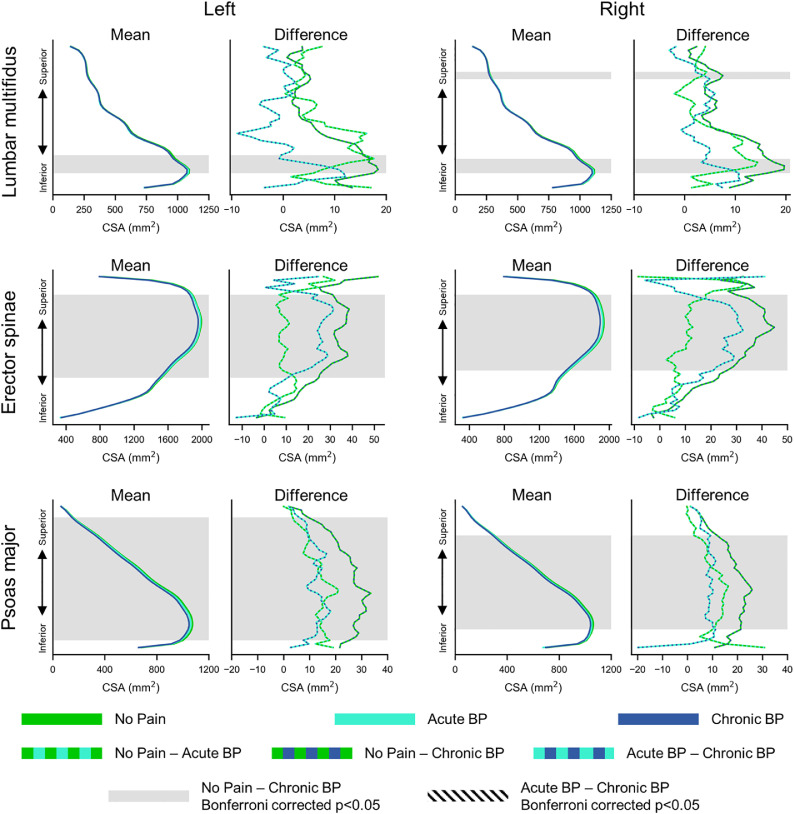
Spatial distribution of cross-sectional area (CSA) along the superior-inferior expanse of the lumbar spine for the left and right lumbar multifidus, erector spinae, and psoas major. Mean CSA (±1 SE error bands) for people with no pain, acute back pain (BP), and chronic BP (Mean) as well as between-group differences (Difference) in CSA are shown. We normalized the CSA measures along the superior-inferior axis such that the inferior most and superior most slices corresponded to 0% and 100% of the lumbar muscle length, respectively. Gray and hatched shading=Bonferroni corrected p-value <.05, showing significant groups differences after family-wise error correction.

The spatial distribution of IMF and CSA varied along the superior-inferior expanse of the lumbar spine and was muscle-specific and consistent across the groups (Fig. 5, Fig. 6). IMF increased inferiorly for the lumbar multifidus and erector spinae while IMF decreased inferiorly for the psoas major. Lumbar multifidus IMF demonstrated a unique sawtooth pattern. The spatial distribution of CSA was also muscle-specific and consistent across the groups. CSA of the lumbar multifidus and psoas major gradually increased inferiorly, peaked in the lower lumbar levels, and then rapidly decreased. CSA of the erector spinae rapidly increased inferiorly, plateaued in the midlumbar levels, and then gradually decreased. The differences in the spatial distribution of IMF and CSA between the groups were not localized to the inferior lumbar levels but more broadly distributed across the lumbar spine for all muscles.

## Discussion

We drew on a large, heterogenous dataset to report on the associations between lumbar paraspinal muscle health and age, BMI, sex, physical activity, and BP (acute and chronic). The findings provide convincing evidence for higher IMF and lower aCSA in BP with the largest differences in those with chronic BP. Next, we assessed paraspinal muscle health along the superior-inferior expanse of the lumbar spine and in contrast to our hypothesis showed that the differences in composition and size in people with BP and no pain were not localized to the inferior lumbar levels but more broadly distributed across the entire lumbar spine.

Information about the associations between lumbar paraspinal muscle health and age, BMI, sex, and physical activity in people with no pain is limited [8,21]. Moreover, these studies typically included small, homogenous samples and used rater-dependent muscle segmentation techniques [10]. Here we first investigated the associations with lumbar paraspinal muscle health and age, sex, BMI, and physical activity in a large, heterogenous dataset of 6,953 participants with no pain using an automated computer-vision model. Our findings are consistent with Crawford et al. (2016) [8] demonstrating that paraspinal muscle health is associated with age and sex with the exception for the association between lumbar multifidus size and age. Significant associations with age and sex may be explained by muscle fiber type distributions. For example, age is related to a reduction in fiber size, that primarily affects type II fibers (ie, fast twitch) not type I fibers (ie, slow twitch) [22]. Additionally, males exhibit greater type II/I fiber ratios while females exhibit greater I/II fiber ratios. Type I muscle fibers appear to express larger magnitudes of IMF, and type II muscle fibers appear to be more susceptible to atrophy in terms of paraspinal muscle health [23]. In contrast to the findings of Crawford et al. (2016) [8], we found significant associations between BMI and paraspinal muscle health. Differences between the findings of both studies are likely to be explained by the sample size (80 versus 6,953 participants with no pain) or methodology as our muscle size measures were corrected for height and measured across the entire muscle between the L1 and L5 vertebral levels. As a step towards developing reference values for research and clinical applications, we provide tables of IMF and aCSA from people with no pain subgrouped by age, BMI, and sex (Supplementary Tables 3 and 4). With further refinement, normative reference values could provide a foundation to clinically assess the magnitude of IMF and aCSA on a patient-by-patient basis with respect to a pain-free reference.

Our findings show an association between paraspinal muscle health and self-reported physical activity. Specifically, higher self-reported physical activity was associated with lower IMF for all muscles and higher aCSA for the erector spinae and psoas major but not the lumbar multifidus. This aligns with the findings from Teichtahl et al. (2015) [24] who also identified an association between physical activity and lumbar multifidus IMF but not size. These findings strengthen the hypothesis that exercise may prevent age-associated increases in IMF, similar to what has been shown for the thigh muscles in older adults [25]. However, due to the cross-sectional design of the study, we cannot make causal inferences. Additionally, the strength of the relationships is relatively small and can be explained by two factors. First, we controlled for age, sex, and BMI, so the strength of the relationship should be interpreted as the variance explained beyond that explained by age, sex, and BMI. Second, despite the wide use of IPAQ [14], the IPAQ is based on self-reported measures of physical activity and has reduced accuracy and reliability to more objective measures of physical activity (eg, accelerometry) and tends to overestimate the time spent in moderate and vigorous physical activity [26,27]. Longitudinal studies, employing more objective measures of physical activity, are needed to more fully delineate and understand the cause-and-effect relationship between physical activity and paraspinal muscle health.

Concerning the magnitude of paraspinal muscle health in BP, we reached similar conclusions as Mengiardi et al. (2006) [28], indicating that people with chronic BP have higher levels of IMF compared to people with no pain. However, the mean differences in IMF between groups in this study were smaller (≤1.6% versus 9.1%). Such disparity may be due to differences in quantification methods (single-voxel MR spectroscopy versus Dixon fat-water MRI). While MR spectroscopy is highly accurate and sensitive to map the metabolic status of muscular tissue [29], considerable sampling error occurs due to user-dependent positioning of the field-of-view [30]. Dixon fat-water MRI is considered the current reference standard for quantifying IMF across an entire muscle [31]. Additionally, the differences between studies can be explained by the increased heterogeneity of the sample due the larger sample size (50 versus 9,564 participants with and without BP) and adjustment for confounders that, in this and other investigations, are related to paraspinal muscle health decline like age, BMI, sex, and physical activity [28]. Next, we showed the magnitude of the differences for both IMF and aCSA between groups were more pronounced in people with chronic BP versus no pain compared to those with acute BP versus no pain. Muscle morphological mechanisms appear to be time-specific after injury [7], which likely explains the larger magnitude changes in lumbar paraspinal IMF and aCSA in chronic BP compared to those with acute BP. Due to the lack of measures on the duration and intensity of BP, we were unable to more finely assess the relationship between paraspinal muscle health and BP. More granular clinical information could transform our understanding of the clinical relevance of lumbar paraspinal muscle health in the development of and maintenance for BP.

In addition to global differences in lumbar paraspinal muscle health, we assessed IMF and CSA spatially along the superior-inferior expanse of the lumbar spine. To date, information about the spatial distribution in health and disease of lumbar paraspinal muscle composition and size is limited [9,32]. We show that the spatial distribution of IMF and aCSA is muscle-specific and consistent across the groups. Interestingly, we identified a unique sawtooth pattern in IMF for the lumbar multifidus. In a secondary analysis, one rater (EW) identified the disc, lower endplate, mid vertebral, and upper endplate levels in the 130 participants with manual segmentations, showing that IMF is higher and lower at the lower and upper endplates levels, respectively (Supplementary Fig. 4). Complementary to Mhuiris et al. (2016) [9], the secondary analysis demonstrates that lumbar multifidus IMF is localized close to the laminae, spinous process, and facet joints [9]. Since the lumbar multifidus IMF varies across the lumbar spine, we recommend assessing IMF across multiple slices to reduce variability in measuring and reporting on IMF. Contradictory to our hypothesis, we found that the differences in paraspinal muscle health in chronic BP versus no pain were not prominent at the inferior lumbar levels where spinal pathology is most common [33] but more broadly distributed across the lumbar spine. The observed global changes may be explained by unloading or disuse-mediated changes (eg, inactivity [6] or pain-related avoidance behavior [34]), which are expected to cause a generalized muscle health decline across the entire muscle [6]. Here, we assessed the spatial distribution of muscle health only along one dimension (ie, the superior-inferior axis). Using a template-based spatial parametric mapping approach, as commonly performed in brain imaging, would permit the exploration of and reporting on the 3D spatial distribution of IMF and its association with age, BMI, sex, and physical activity [35]. Doing so could improve the mechanistic understanding of how paraspinal muscle health affects lumbar spine function.

## Limitations

While the UK Biobank provides a large, heterogeneous sample from the UK, with considerable variability in age, BMI, sex, and physical activity, the cohort does not appear to be fully representative of the UK population [30]. First, a selection bias towards healthy volunteers exists in the UK Biobank, likely resulting in the inclusion of participants with less severe BP compared to the clinical population seeking care for BP [30]. Due to the lack of additional information on BP, we were unable to deeply characterize the sample with respect to BP intensity, BP duration, and BP-related disability or further interrogate the relationship between lumbar paraspinal muscle health and these BP measures. Given the possible selection bias towards less severe BP, the differences in muscle composition and size may be larger in the population of BP patients who are actively seeking care for BP. Second, the ethnic background of participants is predominantly Caucasian (97.2%) and does not represent the diverse ethnicity of the UK or global population [36]. Genetic factors are known to influence muscle composition and size [37], so IMF and aCSA likely vary with ethnic background, and the effects of ethnic background on muscle health need to be interrogated to investigate how the associations with age, sex, BMI, physical activity, and BP generalize to the population as a whole [37]. Third, we excluded 71 participants with poor segmentation quality, of which 25 participants had BP and 20 participants reported no pain. The excluded participants had higher BMI 35.2 (9.8) kg/m^2^ (p<.001) compared to the participants included in this study (26.4 [4.1] kg/m^2^), which may have influenced the results. However, we believe this concern is reduced as the number of excluded participants for poor segmentation quality (n=71) was small relative to the overall sample of 9,564. Lastly, training and testing were limited to the manual segmentations from a single rater. Previously, we reported excellent lumbar paraspinal muscle segmentation interrater reliability (ICC_2,1_≥0.940), mitigating concerns regarding the use of a single rater.

## Conclusions

Paraspinal muscle health is associated with age, BMI, sex, and physical activity with the exception of the association between lumbar multifidus size and age and physical activity. People with BP have higher IMF and lower aCSA than people with no pain (chronic BP>acute BP). The differences were not localized but broadly distributed across the lumbar spine. When interpreting measures of paraspinal muscle health in the research or clinical setting, the associations with age, BMI, sex, and physical activity should be considered.

## Additional information

The authors certify that they have no affiliations with or financial involvement in any organization or entity with a direct financial interest in the subject matter or materials discussed in the article.

## Data sharing statement

We do not have permission to share the data. Researchers can apply to use the UK Biobank resource for health-related research that is in the public interest (https://www.ukbiobank.ac.uk/register-apply/).

## CRediT authorship contribution statement

**Evert Onno Wesselink:** Conceptualization, Formal analysis, Methodology, Software, Writing – original draft, Writing – review & editing, Investigation, Validation, Visualization. **Annelies Pool-Goudzwaard:** Conceptualization, Formal analysis, Methodology, Supervision, Visualization, Writing – original draft, Writing – review & editing, Validation. **Benjamin De Leener:** Resources, Software, Supervision, Writing – review & editing. **Christine Sze Wan Law:** Data curation, Project administration, Resources, Software, Writing – review & editing, Investigation. **Meredith Blair Fenyo:** Data curation, Investigation, Project administration, Resources, Software, Writing – review & editing. **Gabriella Marie Ello:** Data curation, Investigation, Project administration, Resources, Software, Writing – review & editing. **Michel Willem Coppieters:** Conceptualization, Investigation, Supervision, Validation, Visualization, Writing – review & editing. **James Matthew Elliott:** Conceptualization, Methodology, Supervision, Validation, Writing – original draft, Writing – review & editing. **Sean Mackey:** Conceptualization, Data curation, Project administration, Resources, Software, Supervision, Writing – review & editing. **Kenneth Arnold Weber:** Conceptualization, Data curation, Formal analysis, Investigation, Methodology, Project administration, Resources, Software, Supervision, Validation, Visualization, Writing – original draft, Writing – review & editing.

## Declaration of competing interest

One or more of the authors declare financial or professional relationships on ICMJE-TSJ disclosure forms.

## Appendix Supplementary materials

Image, application 1

Image, application 2

Image, application 3

Image, application 4

Image, application 5

Image, application 6

Image, application 7

Image, application 8

Image, application 9

## Footnotes

Supplementary material associated with this article can be found in the online version at https://doi.org/10.1016/j.spinee.2024.02.013.
