# Supplementary material for: Investigating the associations between lumbar paraspinal muscle health and age, BMI, sex, physical activity, and back pain using an automated computer-vision model: a UK Biobank study
Source: Spine J. 2024 Jul;24(7):1253–66. doi: 10.1016/j.spinee.2024.02.013 (PMC11779699; doi:10.1016/j.spinee.2024.02.013)
Supplement: Supplementary file 1 [file mmc1.docx]

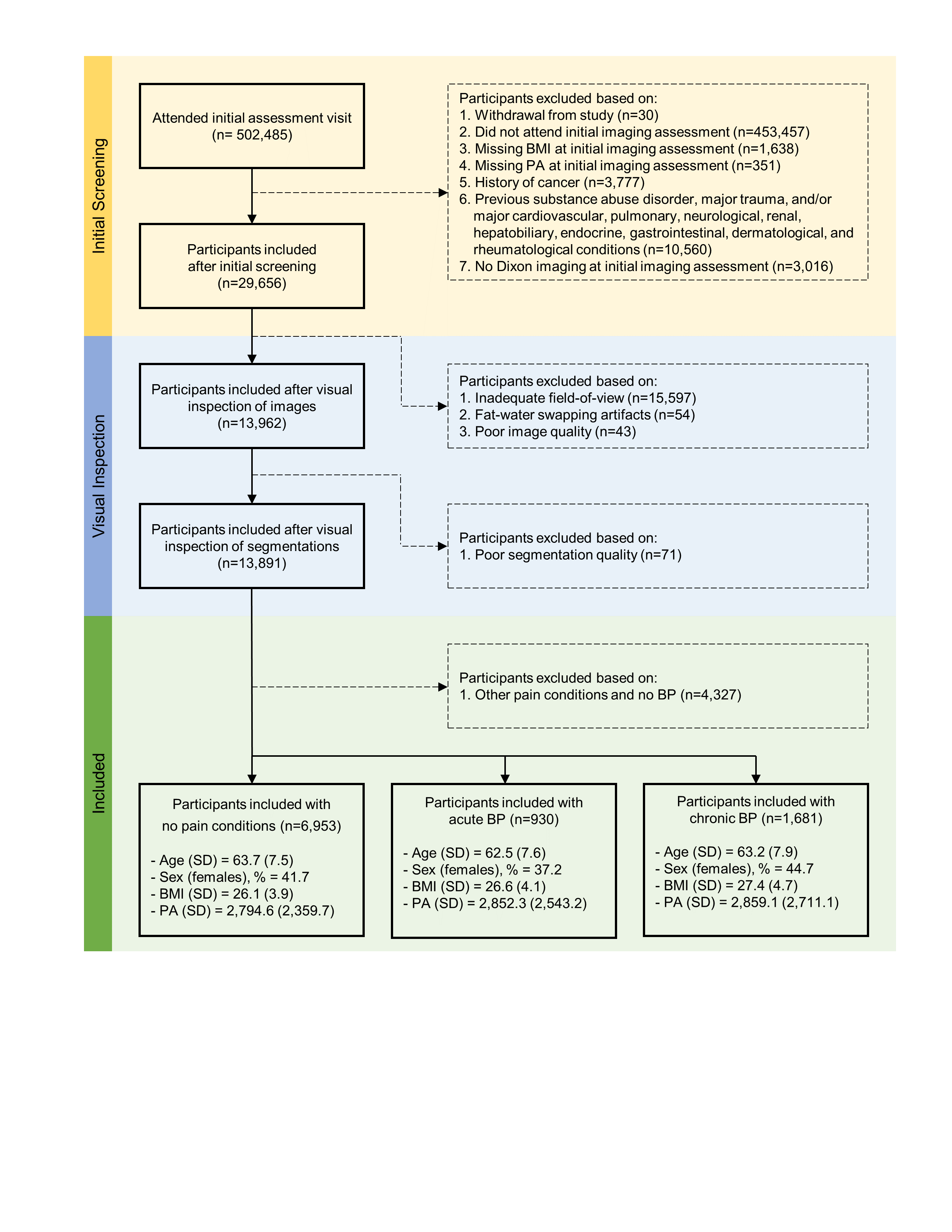


**SUPPLEMENTARY FIGURE 1.** Flowchart for inclusion and exclusion of participants from the UK Biobank. Demographic variables per group (no pain, acute BP and chronic BP) are presented as mean + SD for age, BMI and self-reported physical activity (PA).
