# Supplementary material for: Investigating the associations between lumbar paraspinal muscle health and age, BMI, sex, physical activity, and back pain using an automated computer-vision model: a UK Biobank study
Source: Spine J. 2024 Jul;24(7):1253–66. doi: 10.1016/j.spinee.2024.02.013 (PMC11779699; doi:10.1016/j.spinee.2024.02.013)
Supplement: Supplementary file 2 [file mmc2.docx]

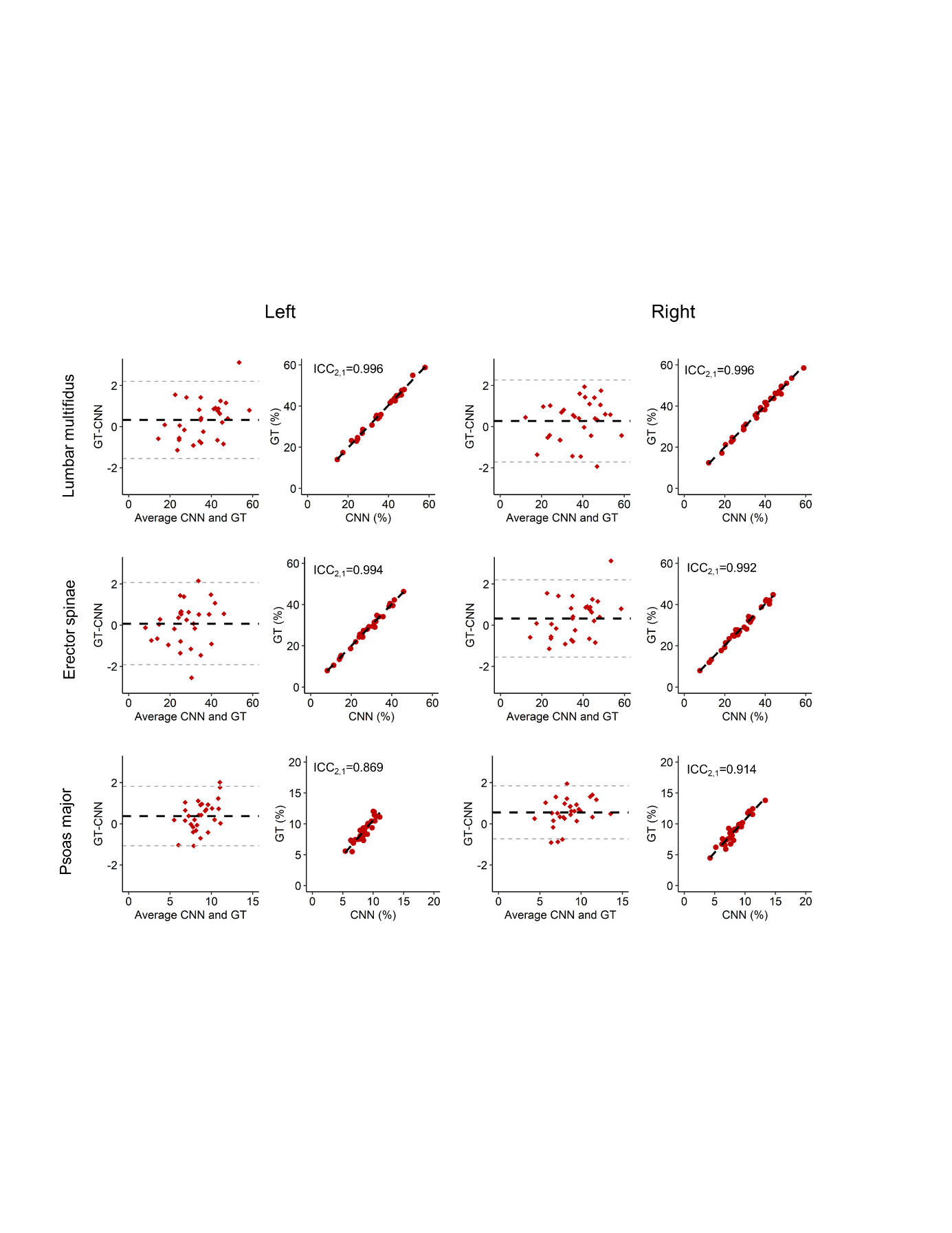


**SUPPLEMENTARY FIGURE 2.** Reliability and accuracy of the Convolutional Neural Network. Bland Altman (black dashed line = mean error, grey dashed lines = 95% limits of agreement) and correlation plots (black dashed line = best fit line) are shown for IMF (in %) of the left and right lumbar paraspinal muscles. *GT*  Ground truth.
