# Supplementary material for: Investigating the associations between lumbar paraspinal muscle health and age, BMI, sex, physical activity, and back pain using an automated computer-vision model: a UK Biobank study
Source: Spine J. 2024 Jul;24(7):1253–66. doi: 10.1016/j.spinee.2024.02.013 (PMC11779699; doi:10.1016/j.spinee.2024.02.013)
Supplement: Supplementary file 4 [file mmc4.docx]

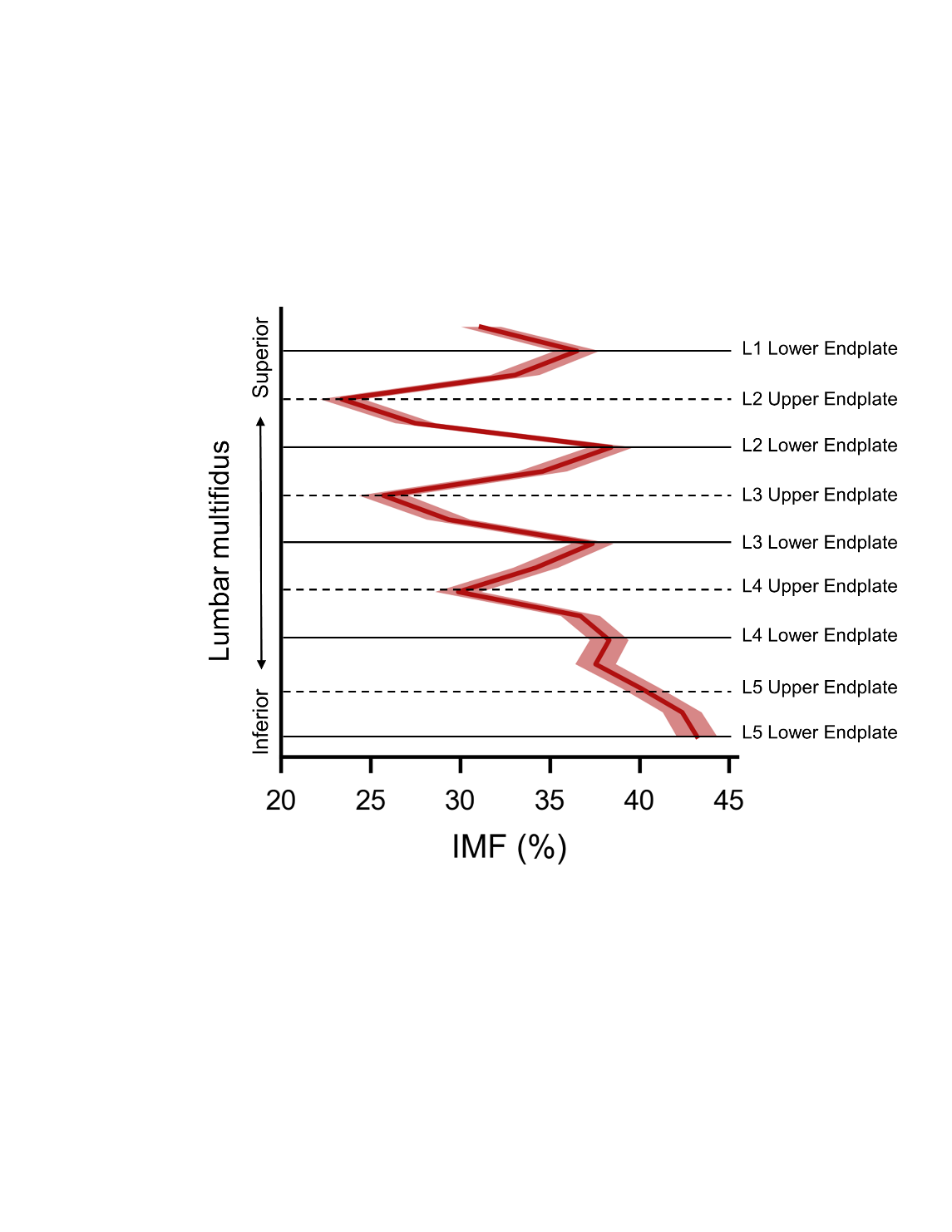


**SUPPLEMENTARY FIGURE 4.** Slice identification after calculating intramuscular fat levels for the lumbar multifidus per level in 130 participants used for CNN training and testing. The total length is normalized from inferior (lower endplate of L5) to superior (mid vertebral level L1). IMF = Intramuscular Fat.
