# Supplementary material for: Investigating the associations between lumbar paraspinal muscle health and age, BMI, sex, physical activity, and back pain using an automated computer-vision model: a UK Biobank study
Source: Spine J. 2024 Jul;24(7):1253–66. doi: 10.1016/j.spinee.2024.02.013 (PMC11779699; doi:10.1016/j.spinee.2024.02.013)
Supplement: Supplementary file 5 [file mmc5.docx]

**SUPPLEMENTARY METHODS**

Automated Assessment of Lumbar Paraspinal Muscle Composition and Size

*Model Training and Testing*

We used a modified 2D U-Net CNN architecture for image segmentation because of good performance in highly anisotropic image data as previously reported[2]. We increased the amount of filter maps (64, 128, 256, 512, and 1,026) to deal with high morphometric muscle variability for low- to mid-level features like edges, contrasts, and shapes. A NVIDIA RTX 3090 24GB graphical processing unit (GPU, NVIDIA, Santa Clara, CA, USA) (spatial window batch size=1, batch size=1, optimizer=AdamW, loss function=DiceCEloss, weight decay=0.0001, and learning rate=0.001) was used for model training. The images were randomly cropped to a spatial window size with the centre being a foreground or background voxel based on a positive/negative ratio of one. The 2D model was trained on axial slices using a spatial window size of 160×160×1. The model was initialized with random weights using equivalent randomizations, and the deterministic seed was set to zero. One blinded rater (EW) with extensive training in lumbar spine anatomy and imaging manually segmented the muscles of interest (i.e., left and right lumbar multifidus, erector spinae, and psoas major) from a dataset of 65 participants with no pain and 65 participants with chronic BP using anatomical cross-references as previously described[3]. In addition, the images were resampled to 2.23mm × 2.23mm x 4.50mm, and the range of pixel values were normalized per participant to generate an equivalent standard intensity scale between the images. All voxels in the image were normalized by subtracting to the mean intensity of all voxels per subject divided by the standard deviation of all voxels per subject. Data augmentation was used to increase the variability in the training images[4]. Affine transformations were applied to the training scheme with a probability of 0.4, including scaling (-2.5–2.5%), rotation (x=-2.5 – 2.5°, y=-2.5 – 2.5°, z=-2.5 – 2.5°) and translation (in voxels relative to the centre of the input image, x=-25 – 25 voxels, y=-25 – 25 voxels, z=-2 – 2 voxels). These specific augmentation hyperparameters were chosen to mimic variations in positioning on the scanner bed and to prevent the network from fixating on specific regions of its perceptive field[5, 6].The images were smart-cached to the RAM to improve training speed. Data augmentation, model training, and model testing were performed using MONAI, an open-source community supported, Pytorch-based framework for deep learning in healthcare imaging[7].

CNN performance was evaluated on the testing dataset using the Sørensen-Dice index, the Jaccard index, conformity index, true positive rate, true negative rate, positive predictive value, and volume ratio. CNN performance was also calculated for IMF and aCSA using the mean absolute error (MAE), root squared mean error (RSME), coefficient of determination (R^2^) and the intra-class coefficient (two-way random effect, absolute agreement for single rater; ICC_2,1_). Results were visually presented as correlation and Bland-Altman plots.
