# Supplementary material for: Investigating the associations between lumbar paraspinal muscle health and age, BMI, sex, physical activity, and back pain using an automated computer-vision model: a UK Biobank study
Source: Spine J. 2024 Jul;24(7):1253–66. doi: 10.1016/j.spinee.2024.02.013 (PMC11779699; doi:10.1016/j.spinee.2024.02.013)
Supplement: Supplementary file 6 [file mmc6.docx]

**SUPPLEMENTARY TABLE 1.** Convolutional Neural Network performance. Data is presented as mean + SD

| Muscle | Side | Dice | JI | CC | TPR | TNR | PPV | VR |
| --- | --- | --- | --- | --- | --- | --- | --- | --- |
| Lumbar multifidus | Left | 0.892 (0.02) | 0.806 (0.04) | 0.756 (0.06) | 0.900 (0.03) | 1.000 (0.00) | 0.885 (0.03) | 1.019 (0.06) |
|  | Right | 0.898 (0.02) | 0.815 (0.02) | 0.772 (0.04) | 0.908 (0.03) | 1.000 (0.00) | 0.889 (0.03) | 1.024 (0.05) |
| Erector spinae | Left | 0.916 (0.02) | 0.846 (0.03) | 0.817 (0.05) | 0.928 (0.03) | 0.999 (0.00) | 0.906 (0.03) | 1.024 (0.05) |
|  | Right | 0.912 (0.01) | 0.838 (0.02) | 0.806 (0.04) | 0.918 (0.03) | 0.999 (0.00) | 0.907 (0.03) | 1.013 (0.05) |
| Psoas major | Left | 0.917 (0.02) | 0.848 (0.04) | 0.818 (0.05) | 0.932 (0.02) | 1.000 (0.00) | 0.906 (0.04) | 1.028 (0.06) |
|  | Right | 0.918 (0.02) | 0.848 (0.03) | 0.820 (0.05) | 0.930 (0.04) | 1.000 (0.00) | 0.905 (0.03) | 1.031 (0.05) |

Dice = Sørensen-Dice index, JI = Jaccard Index, CC = Conformity Index, TPR = True Positive Rate, TNR = True Negative Rate, PPV = Positive Predictive value, VR = Volume Ratio.
