# Supplementary material for: Investigating the associations between lumbar paraspinal muscle health and age, BMI, sex, physical activity, and back pain using an automated computer-vision model: a UK Biobank study
Source: Spine J. 2024 Jul;24(7):1253–66. doi: 10.1016/j.spinee.2024.02.013 (PMC11779699; doi:10.1016/j.spinee.2024.02.013)
Supplement: Supplementary file 7 [file mmc7.docx]

**SUPPLEMENTARY TABLE 2.**  Accuracy and reliability of the Convolutional Neural Network for intramuscular fat (%) and cross-sectional area.

| Intramuscular fat (%) | | | | | | | | | | |
| --- | --- | --- | --- | --- | --- | --- | --- | --- | --- | --- |
| Muscle | Side | Mean | Bias | 95% LOA | MAE | RMSE | R^2^ | ICC_2,1_ | 95% CI | p |
| Lumbar multifidus | Left | 36.0 (2.0) | -0.3 | -2.20 - 1.54 | 0.810 | 0.995 | 0.991 | 0.996 | 0.991-0.998 | <0.001 |
|  | Right | 37.3 (2.0) | -0.3 | -2.26 - 1.71 | 0.897 | 1.036 | 0.991 | 0.996 | 0.991-0.998 | <0.001 |
| Erector spinae | Left | 27.4 (1.7) | -0.1 | -2.06 -1.91 | 0.807 | 1.000 | 0.988 | 0.994 | 0.988-0.997 | <0.001 |
|  | Right | 28.1 (1.7) | -0.3 | -2.54 - 1.93 | 0.919 | 1.160 | 0.983 | 0.992 | 0.983-0.996 | <0.001 |
| Psoas major | Left | 8.7 (0.3) | -0.4 | -1.82 - 1.07 | 0.655 | 0.814 | 0.645 | 0.869 | 0.700-0.940 | <0.001 |
|  | Right | 8.5 (0.4) | -0.6 | -1.84 - 0.73 | 0.736 | 0.852 | 0.795 | 0.914 | 0.625-0.970 | <0.001 |
| CSA (ml) | | | | | | | | | | |
| Muscle | Side | Mean | Bias | 95% LOA | MAE | RMSE | R^2^ | ICC_2,1_ | 95% CI | p |
| Lumbar multifidus | Left | 95.0 (3.0) | 1.5 | -8.52 - 11.63 | 4.038 | 5.289 | 0.892 | 0.949 | 0.894-0.975 | <0.001 |
|  | Right | 99.2 (3.2) | 2.2 | -8.32 - 12.61 | 4.536 | 5.672 | 0.897 | 0.950 | 0.890-0.977 | <0.001 |
| Erector spinae | Left | 263.1 (13.6) | 6.5 | -17.35 - 30.36 | 11.32 | 13.621 | 0.967 | 0.984 | 0.956-0.993 | <0.001 |
|  | Right | 257.6 (13.7) | 3.0 | -21.83 - 27.93 | 11.39 | 12.848 | 0.970 | 0.985 | 0.970-0.993 | <0.001 |
| Psoas major | Left | 111.3 (7.6) | 1.9 | -14.02 - 17.78 | 5.513 | 8.196 | 0.957 | 0.981 | 0.961-0.991 | <0.001 |
|  | Right | 110.3 (8.2) | 2.6 | -7.44 - 12.74 | 4.285 | 5.711 | 0.983 | 0.992 | 0.979-0.996 | <0.001 |

MAE = Mean Absolute Error, RMSE = Root Mean Square Error, LOA = Limits of Agreement
