# Supplementary material for: Investigating the associations between lumbar paraspinal muscle health and age, BMI, sex, physical activity, and back pain using an automated computer-vision model: a UK Biobank study
Source: Spine J. 2024 Jul;24(7):1253–66. doi: 10.1016/j.spinee.2024.02.013 (PMC11779699; doi:10.1016/j.spinee.2024.02.013)
Supplement: Supplementary file 8 [file mmc8.docx]

**SUPPLEMENTARY TABLE 3.**  Intramuscular fat (mean ± SD) by muscle, age, BMI, and sex in 6,953 participants with no pain.

| BMI < 18.5 | | | | | | | | | | | |
| --- | --- | --- | --- | --- | --- | --- | --- | --- | --- | --- | --- |
| Males | | | | | | | Females | | | | |
| Age | | 40-49 | 50-59 | 60-69 | 70-79 | 80-89 | 40-49 | 50-59 | 60-69 | 70-79 | 80-89 |
| n | | 0 | 3 | 3 | 4 | 0 | 1 | 12 | 19 | 6 | 0 |
| Lumbar multifidus | Left | - | 19.7(3.5) | 34.1(8.0) | 36.4(9.2) | - | 28.8(0.0) | 34.8(5.1) | 38.8(7.4) | 39.9(5.2) | - |
|  | Right | - | 22.0(4.6) | 33.2(7.6) | 36.0(8.5) | - | 28.3(0.0) | 34.7(5.2) | 37.7(6.2) | 38.0(4.5) | - |
| Erector spinae | Left | - | 12.2(2.9) | 26.9(11.2) | 19.7(3.4) | - | 18.9(0.0) | 23.7(3.7) | 27.9(5.5) | 31.7(5.4) | - |
|  | Right | - | 11.7(1.7) | 25.6(8.4) | 19.5(3.0) | - | 18.1(0.0) | 22.8(4.7) | 26.1(4.2) | 27.6(5.9) | - |
| Psoas Major | Left | - | 4.8(0.2) | 7.9(2.4) | 5.7(0.6) | - | 5.7(0.0) | 5.8(0.8) | 6.2(1.0) | 6.8(0.9) | - |
|  | Right | - | 4.5(0.3) | 7.1(2.1) | 4.9(0.2) | - | 4.8(0.0) | 5.1(0.5) | 5.4(0.8) | 5.6(0.5) | - |
| BMI 18.5 – 24.9 | | | | | | | | | | | |
| Males | | | | | | | Females | | | | |
| Age | | 40-49 | 50-59 | 60-69 | 70-79 | 80-89 | 40-49 | 50-59 | 60-69 | 70-79 | 80-89 |
| n | | 20 | 339 | 637 | 447 | 8 | 34 | 524 | 650 | 258 | 5 |
| Lumbar multifidus | Left | 25.6(7.7) | 25.6(6.3) | 29.8(7.3) | 35.0(8.3) | 35.6(6.3) | 30.6(7.2) | 35.5(7.7) | 40.9(7.7) | 48.8(7.5) | 47.1(7.0) |
|  | Right | 25.5(7.0) | 25.9(6.5) | 30.1(7.2) | 35.1(8.3) | 34.5(4.9) | 30.5(6.7) | 35.7(7.7) | 41.2(7.8) | 45.8(7.6) | 45.0(5.8) |
| Erector spinae | Left | 14.9(5.0) | 16.7(4.8) | 20.6(6.2) | 25.1(7.7) | 27.0(7.2) | 22.6(6.1) | 26.5(6.8) | 31.0(7.0) | 35.3(7.3) | 36.9(7.6) |
|  | Right | 15.5(5.1) | 16.8(5.0) | 20.9(6.4) | 25.2(7.4) | 28.8(7.5) | 21.8(5.2) | 25.9(6.9) | 31.1(7.2) | 35.0(7.3) | 35.9(6.7) |
| Psoas major | Left | 6.6(1.3) | 6.8(1.2) | 7.3(1.2) | 7.7(1.4) | 8.2(1.3) | 6.6(0.9) | 7.3(1.3) | 7.9(1.3) | 8.5(1.4) | 8.8(1.4) |
|  | Right | 6.0(1.4) | 6.2(1.2) | 6.7(1.3) | 7.2(1.6) | 7.6(2.2) | 5.8(1.0) | 6.4(1.2) | 6.9(1.4) | 7.5(1.5) | 7.1(0.7) |
| BMI 25.0 – 29.9 | | | | | | | | | | | |
| Males | | | | | | | Females | | | | |
| Age | | 40-49 | 50-59 | 60-69 | 70-79 | 80-89 | 40-49 | 50-59 | 60-69 | 70-79 | 80-89 |
| n |  | 45 | 474 | 846 | 601 | 12 | 27 | 311 | 469 | 202 | 2 |
| Lumbar multifidus | Left | 25.0(7.0) | 26.8(6.8) | 31.9(8.0) | 36.8(8.6) | 39.0(5.7) | 33.2(4.8) | 37.4(7.6) | 43.6(8.2) | 47.4(8.0) | 55.1(12.4) |
|  | Right | 25.8(6.6) | 27.2(6.9) | 32.4(8.0) | 37.8(8.5) | 40.7(7.5) | 33.9(5.2) | 38.1(7.6) | 44.0(8.5) | 48.2(8.3) | 54.7(7.2) |
| Erector spinae | Left | 17.1(4.5) | 18.3(5.6) | 22.8(7.1) | 27.4(7.6) | 32.2(8.3) | 25.8(5.1) | 28.8(6.7) | 33.9(7.7) | 37.5(8.0) | 50.1(8.5) |
|  | Right | 17.4(4.1) | 18.9(5.7) | 23.4(6.9) | 28.3(7.6) | 33.1(9.4) | 26.0(5.5) | 28.7(6.8) | 34.0(7.7) | 37.7(7.8) | 47.6(4.4) |
| Psoas major | Left | 7.5(1.2) | 7.3(1.2) | 7.9(1.5) | 8.4(1.8) | 8.6(1.3) | 8.0(0.8) | 8.2(1.1) | 8.5(1.1) | 8.9(1.3) | 9.4(2.4) |
|  | Right | 7.2(1.3) | 7.2(1.3) | 7.8(1.7) | 8.5(1.9) | 8.5(1.5) | 7.4(1.0) | 7.5(1.2) | 7.9(1.2) | 8.4(1.4) | 7.9(1.5) |
| BMI ≥ 30.0 | | | | | | | | | | | |
| Males | | | | | | | Females | | | | |
| Age | | 40-49 | 50-59 | 60-69 | 70-79 | 80-89 | 40-49 | 50-59 | 60-69 | 70-79 | 80-89 |
| n |  | 17 | 168 | 282 | 145 | 2 | 3 | 130 | 172 | 75 | 0 |
| Lumbar multifidus | Left | 27.4(6.9) | 28.3(6.9) | 34.2(8.3) | 39.8(8.7) | 45.5(2.1) | 36.9(5.4) | 39.5(7.5) | 45.9(8.2) | 51.5(8.2) | - |
|  | Right | 29.5(7.5) | 29.2(7.3) | 35.2(8.3) | 41.4(8.7) | 48.6(11.6) | 33.6(4.0) | 40.1(7.3) | 46.7(8.1) | 52.1(8.5) | - |
| Erector spinae | Left | 20.8(6.2) | 20.0(5.6) | 26.1(7.9) | 31.1(9.3) | 36.5(14.8) | 26.9(1.6) | 31.3(6.4) | 35.9(8.2) | 40.6(8.3) | - |
|  | Right | 22.3(6.6) | 21.4(6.0) | 27.4(7.9) | 32.8(9.6) | 35.1(13.0) | 28.7(5.4) | 31.7(6.4) | 36.7(8.2) | 41.2(8.6) | - |
| Psoas major | Left | 8.1(1.5) | 7.8(1.4) | 8.7(2.0) | 9.7(2.6) | 11.0(4.8) | 8.2(0.3) | 8.3(1.2) | 8.7(1.2) | 9.1(1.3) | - |
|  | Right | 8.0(1.7) | 8.0(1.4) | 9.1(2.0) | 10.2(2.7) | 9.6(3.7) | 8.5(2.1) | 8.2(1.4) | 8.6(1.4) | 9.3(1.9) | - |
