# Supplementary material for: Investigating the associations between lumbar paraspinal muscle health and age, BMI, sex, physical activity, and back pain using an automated computer-vision model: a UK Biobank study
Source: Spine J. 2024 Jul;24(7):1253–66. doi: 10.1016/j.spinee.2024.02.013 (PMC11779699; doi:10.1016/j.spinee.2024.02.013)
Supplement: Supplementary file 9 [file mmc9.docx]

**SUPPLEMENTARY TABLE 4.**  Average CSA (mean ± SD) by muscle, age, BMI, and sex in 6,953 participants with no pain. Average CSA is calculated by dividing the total CSA by the number of segmented slices.

| BMI < 18.5 | | | | | | | | | | | |
| --- | --- | --- | --- | --- | --- | --- | --- | --- | --- | --- | --- |
| Males | | | | | | | Females | | | | |
| Age | | 40-49 | 50-59 | 60-69 | 70-79 | 80-89 | 40-49 | 50-59 | 60-69 | 70-79 | 80-89 |
| n | | 0 | 3 | 3 | 4 | 0 | 1 | 12 | 19 | 6 | 0 |
| Lumbar multifidus | Left | - | 535.6(64.0) | 525.0(47.7) | 515.8(52.7) | - | 479.6(0.0) | 468.7(66.0) | 478.0(62.3) | 450.0(58.5) | - |
|  | Right | - | 577.0(71.9) | 537.9(63.7) | 525.2(62.9) | - | 500.4(0.0) | 472.5(63.2) | 474.6(67.1) | 448.8(52.6) | - |
| Erector spinae | Left | - | 1618.1(73.4) | 1319.0(181.4) | 1266.5(197.0) | - | 1298.8(0.0) | 1202.2(109.6) | 1185.1(128.6) | 1157.4(156.9) | - |
|  | Right | - | 1393.0(79.7) | 1281.4(141.4) | 1262.8(182.9) | - | 1301.6(0.0) | 1124.5(112.6) | 1102.0(137.1) | 1075.5(85.3) | - |
| Psoas major | Left | - | 814.0(200.0) | 553.7(54.7) | 579.8(183.6) | - | 483.5(0.0) | 461.4(92.7) | 418.4(74.2) | 364.0(81.4) | - |
|  | Right | - | 771.7(182.6) | 533.1(24.9) | 522.0(145.1) | - | 405.4(0.0) | 439.5(78.7) | 403.6(68.1) | 363.9(87.8) | - |
| BMI 18.5 – 24.9 | | | | | | | | | | | |
| Males | | | | | | | Females | | | | |
| Age | | 40-49 | 50-59 | 60-69 | 70-79 | 80-89 | 40-49 | 50-59 | 60-69 | 70-79 | 80-89 |
| n | | 20 | 339 | 637 | 447 | 8 | 34 | 524 | 650 | 258 | 5 |
| Lumbar multifidus | Left | 574.4(94.5) | 579.4(74.2) | 588.2(73.8) | 580.6(75.7) | 550.8(52.6) | 557.9(80.5) | 542.3(71.2) | 545.2(73.5) | 541.7(70.7) | 560.2(113.6) |
|  | Right | 587.6(92.4) | 591.6(75.5) | 599.3(80.6) | 592.0(81.3) | 568.2(81.0) | 573.4(81.8) | 552.8(71.4) | 558.1(78.7) | 555.5(73.1) | 589.0(133.1) |
| Erector spinae | Left | 1683.8(226.0) | 1700.3(188.8) | 1628.7(192.1) | 1564.5(191.2) | 1447.0(157.8) | 1450.9(228.4) | 1364.1(147.4) | 1332.9(163.3) | 1293.9(152.7) | 1331.9(95.5) |
|  | Right | 1645.6(193.9) | 1657.8(184.6) | 1588.3(188.9) | 1529.6(194.3) | 1443.6(180.3) | 1401.4(259.3) | 1306.8(151.5) | 1283.4(167.1) | 1245.8(147.2) | 1281.2(149.3) |
| Psoas major | Left | 924.7(150.1) | 885.0(150.3) | 815.7(144.8) | 733.3(135.7) | 668.1(67.2) | 592.1(118.7) | 541.3(104.5) | 496.1(87.7) | 461.2(90.5) | 390.2(53.2) |
|  | Right | 860.6(125.3) | 831.6(139.4) | 770.6(135.9) | 690.6(124.0) | 601.3(51.7) | 543.3(112.1) | 501.4(89.6) | 464.8(81.0) | 441.1(85.5) | 416.4(30.5) |
| BMI 25.0 – 29.9 | | | | | | | | | | | |
| Males | | | | | | | Females | | | | |
| Age | | 40-49 | 50-59 | 60-69 | 70-79 | 80-89 | 40-49 | 50-59 | 60-69 | 70-79 | 80-89 |
| n | | 45 | 474 | 846 | 601 | 12 | 27 | 311 | 469 | 202 | 2 |
| Lumbar multifidus | Left | 629.1(66.7) | 613.5(76.2) | 619.9(77.9) | 614.3(75.6) | 602.4(84.1) | 562.7(92.6) | 562.3(66.2) | 565.5(73.2) | 561.0(70.8) | 429.7(6.7) |
|  | Right | 645.1(73.6) | 628.9(81.0) | 628.4(81.3) | 623.7(80.5) | 603.6(76.7) | 580.3(98.3) | 569.8(67.7) | 576.8(78.6) | 573.0(77.2) | 473.0(14.1) |
| Erector spinae | Left | 1910.1(246.6) | 1882.2(225.9) | 1813.9(212.4) | 1737.5(203.8) | 1659.3(249.3) | 1492.9(173.0) | 1471.7(160.5) | 1425.0(158.0) | 1400.7(160.0) | 1039.0(68.0) |
|  | Right | 1887.8(200.5) | 1848.4(211.5) | 1782.6(207.7) | 1705.3(203.2) | 1577.4(189.9) | 1467.7(181.0) | 1426.7(157.8) | 1384.4(150.3) | 1371.9(156.0) | 1032.7(20.1) |
| Psoas major | Left | 905.3(136.7) | 930.3(163.3) | 846.4(154.9) | 773.9(147.1) | 777.3(154.2) | 559.3(118.3) | 542.5(95.8) | 516.5(94.7) | 494.4(99.2) | 470.2(168.8) |
|  | Right | 877.9(143.1) | 903.0(151.4) | 812.0(147.7) | 757.7(131.6) | 718.6(142.9) | 535.8(97.5) | 524.5(89.4) | 502.6(89.1) | 477.0(91.8) | 406.6(120.6) |
| BMI ≥ 30.0 | | | | | | | | | | | |
| Males | | | | | | | Females | | | | |
| Age | | 40-49 | 50-59 | 60-69 | 70-79 | 80-89 | 40-49 | 50-59 | 60-69 | 70-79 | 80-89 |
| n | | 17 | 168 | 282 | 145 | 2 | 3 | 130 | 172 | 75 | 0 |
| Lumbar multifidus | Left | 639.8(58.6) | 644.5(70.7) | 654.9(78.8) | 655.7(73.6) | 749.8(32.2) | 584.0(94.3) | 590.5(74.8) | 599.1(82.9) | 588.9(76.8) | - |
|  | Right | 670.3(90.6) | 660.0(74.2) | 661.6(85.1) | 658.8(76.2) | 734.6(6.0) | 580.0(103.3) | 596.4(77.7) | 598.0(85.4) | 591.8(79.2) | - |
| Erector spinae | Left | 2041.1(236.7) | 2029.0(246.2) | 1957.7(220.9) | 1866.7(222.3) | 1855.3(0.5) | 1646.6(91.0) | 1586.7(191.3) | 1566.6(198.3) | 1526.8(175.2) | - |
|  | Right | 1948.3(178.6) | 1987.6(233.1) | 1951.0(217.7) | 1835.1(216.6) | 1763.8(144.4) | 1569.0(86.1) | 1566.4(190.7) | 1536.4(189.4) | 1508.3(184.9) | - |
| Psoas major | Left | 1021.6(214.8) | 952.2(172.2) | 870.8(148.2) | 800.4(145.9) | 870.9(47.2) | 631.0(97.6) | 572.5(99.7) | 518.3(99.9) | 489.8(93.7) |  |
|  | Right | 975.0(169.4) | 930.1(172.7) | 852.6(137.5) | 799.0(140.5) | 861.6(1.4) | 664.5(90.1) | 572.9(103.1) | 518.5(87.3) | 486.1(84.7) | - |
